# Loss of Parvalbumin in the Hippocampus of MAM Schizophrenia Model Rats Is Attenuated by Peripubertal Diazepam

**DOI:** 10.1093/ijnp/pyw065

**Published:** 2016-07-18

**Authors:** Yijuan Du, Anthony A. Grace

**Affiliations:** Departments of Neuroscience (Dr Du, Dr Grace), Psychiatry (Dr Grace), and Psychology (Dr Grace), University of Pittsburgh, Pittsburgh, PA.

**Keywords:** schizophrenia, animal model, diazepam, parvalbumin interneuron

## Abstract

**Background:**

Loss of parvalbumin interneurons in the hippocampus is a robust finding in schizophrenia brains. Rats exposed during embryonic day 17 to methylazoxymethanol acetate exhibit characteristics consistent with an animal model of schizophrenia, including decreased parvalbumin interneurons in the ventral hippocampus. We reported previously that peripubertal administration of diazepam prevented the emergence of pathophysiology in adult methylazoxymethanol acetate rats.

**Methods:**

We used an unbiased stereological method to examine the impact of peripubertal diazepam treatment on parvalbumin interneuron number in the ventral subiculum, dentate gyrus of the hippocampus and the basolateral amygdala.

**Results:**

Methylazoxymethanol acetate rats with peripubertal diazepam showed significantly more parvalbumin interneurons (3355±173 in the ventral subiculum, 1211±76 in the dentate gyrus) than methylazoxymethanol acetate without diazepam (2375±109 and 824±54, respectively). No change was found in the basolateral amygdala.

**Conclusions:**

Peripubertal diazepam attenuated the decrease of parvalbumin in the ventral hippocampus of methylazoxymethanol acetate rats.

## Introduction

Loss of parvalbumin interneurons is one of the most robust findings from postmortem brains of schizophrenia patients (Volk and Lewis, 2002; Zhang and Reynolds, 2002; Konradi et al., 2011; Wang et al., 2011). These fast-spiking, parvalbumin-expressing interneurons are required for gamma oscillations (Sohal et al., 2009) and impact multiple cognitive functions, both of which are disrupted in schizophrenia patients (Uhlhaas and Singer, 2015). Loss of parvalbumin interneurons in the hippocampus and prefrontal cortex has been reported in many animal models of schizophrenia, including neonatal hippocampal lesion (Cabungcal et al., 2014), maternal immune activation (Piontkewitz et al., 2012), amygdala disinhibition (Berretta et al., 2009), and MAM-E17 models (Penschuck et al., 2006; Lodge et al., 2009; Chen et al., 2014; Gill and Grace, 2014).

Rats exposed during embryonic day 17 (E17) to a mitotoxin, methylazoxymethanol acetate (MAM), exhibit behavioral, pharmacological, and anatomical characteristics consistent with an animal model of schizophrenia (Lodge and Grace, 2009). These rats exhibit increases in dopamine neuron population activity, which are thought to underlie the enhanced locomotor response to amphetamine and other psychostimulants (Moore et al., 2006) and are driven by pathologically excessive activity of the hippocampus (Lodge and Grace, 2007). Hippocampal hyperactivity in turn correlates with decreased density of parvalbumin interneurons (Lodge et al., 2009). This loss of parvalbumin interneurons in the hippocampus of MAM rats begins while juveniles and persists into adulthood (Chen et al., 2014; Gill and Grace, 2014).

Peripubertal administration of diazepam (PD31-40) prevents the emergence of the hyperdopaminergic state in adult MAM rats by alleviating the heightened level of anxiety during the peripubertal period (Du and Grace, 2013). However, the cellular mechanism by which this protection occurs is unclear. Parvalbumin interneurons, especially those in the hippocampus, are sensitive to chronic stress (Czeh et al., 2015; Hu et al., 2010). Therefore, one possibility is that this treatment to alleviate stress could protect parvalbumin interneurons from further loss. In this study, we investigated whether peripubertal administration of diazepam can prevent the loss of parvalbumin interneurons in MAM rats.

In addition to alterations in the hippocampus, hyperactivity of the basolateral amygdala (BLA) was observed in both peripubertal and adult MAM rats (Du and Grace, 2016). The BLA also contains a high density of parvalbumin interneurons, innervating and regulating the firing of projection neurons. Therefore, we also examined whether a reduction in parvalbumin interneurons in the BLA contributed to its hyperactivity.

## Methods

### Animals

All procedures were conducted in accordance with the Guide for the Care and Use of Laboratory Animals by the United States Public Health Service and approved by the University of Pittsburgh Institutional Animal Care and Use Committee. Pregnant Sprague-Dawley dams were obtained from Harlan on gestational day (GD) 15 and administered MAM (20mg/kg, i.p., Midwest Research Institute, Kansas City, MO) or saline (Sal) on GD 17. Litters were weaned on postnatal day 23 and housed 2 to 3/cage. Only male offspring were used. Animals were housed in a normal light cycle (lights on 7 am to 7 pm).

### Oral Administration of Diazepam

Diazepam (2-mg tablets, Watson Laboratories, Inc., Corona, CA) was ground to powder and mixed with sweetened condensed milk (Eagle Brand), sucrose powder, and ground mini Nilla Wafers (Kraft Food). Diazepam mixture (5mg/kg) or the same mixture without diazepam was administered during the peripubertal period with once-daily administration on 10 consecutive days (PD31-40), as described previously (Du and Grace, 2013, 2016). The oral administration route was chosen because it is less stressful than i.p. injections, especially for rats at the peripubertal age that are more sensitive to stress, as well as better mimicking the preferred route of drug administration to patients.

### Tissue Preparation

Adult rats (PD83) were anesthetized with sodium pentobarbital (60mg/kg, i.p.) and perfused transcardially with saline followed by 4% paraformaldehyde in 0.1M phosphate buffer (PB). Brains were removed, fixed in 4 % paraformaldehyde for 30 minutes, and stored in 0.1M PB. Before slicing, sections were cryoprotected in 25% sucrose in 0.1M PB for 48 hours and sliced into 60-μm coronal sections. Every fourth section throughout the BLA, ventral subiculum (vSub), and dentate gyrus (DG) of the hippocampus was used for immunohistochemistry, as a random series. Adjacent sections were used for Cresyl violet staining. A total of 9 to 11 sections for Sal:Veh, 8 to 10 for Sal:DZ, 8 to 9 for MAM:Veh, and 9 to 10 for MAM:DZ were used throughout the vSub; 6 sections for Sal:veh and Sal:DZ and 5 to 6 for MAM:Veh and MAM:DZ were from DG. A total of 12 to 14 sections for Sal and 13 to 16 for MAM were from the BLA.

### Immunohistochemistry

Free-floating sections were rinsed in 0.01M PBS and treated with 0.5% sodium borohydride for 30 minutes and 1% hydrogen peroxide. Sections were blocked for 1 hour with 3% normal goat serum and 0.5% Triton in 0.01M PBS and incubated with primary antibody (rabbit polyclonal anti-parvalbumin, 1:5000, PV27, Swant) in the same blocking solution overnight at room temperature. Sections were then incubated in biotinylated secondary antibody (goat anti-rabbit, 1:500, Vector) for 1 hour and then in VECTASTAIN Standard ABC Kit for 40 minutes. Following TBS rinses, the immunoperoxidase reaction was performed in 3’3’-diaminobenzidene solution containing 0.1% hydrogen peroxide for 5 minutes. Sections were mounted on gelatin-coated glass slides; dehydrated through 50%, 75%, 95%, and 100% alcohol and xylene; and coverslipped with permount.

### Stereology

Parvalbumin positive cells were counted in the right BLA, vSub, and DG using an unbiased stereological method: optical fractionator (West et al., 1991). The optical fractionator method provides unbiased estimates of neuron numbers, which are free of assumptions about neuron size and shape.

Sections were examined using a Nikon microscope with a motorized stage (Ludl Electronic Products, Hawthorne, NY) and a digital camera (QImage), using StereoInvestigator 9 software (MicroBrightfield, Inc., Williston, VT). Researchers were blind to treatment of animals. Boundaries of regions of interest were traced on cresyl violet-stained sections, modified on peroxidase-stained sections under 2.5× objective lens, and refined under 10×. Counting was performed using a 60× objective. For the BLA, a 200-μm * 200-μm counting frame was used in a grid size of 325 μm * 325 μm; for vSub, a 150-μm * 150-μm counting frame was used in a grid size of 300 μm * 300 μm; and for ventral DG, a 250-μm * 250-μm counting frame was used in a grid size of 300 μm * 300 μm. A 12-μm height was chosen for counting, with 3 μm for the top guard zone and the rest as bottom guard zone. The coefficient of error Gundersen (m=0), which describes the accuracy of the optical fractionator method used, ranged from 0.07 to 0.17 (average 0.10) for vSub quantification, 0.09 to 0.18 (average 0.12) for ventral DG, 0.07 to 0.12 (average 0.08) for BLA.

The BLA comprises the lateral, basolateral, and the basomedial nuclei. Boundaries of the BLA were defined according to an atlas of the rat brain (Paxinos and Watson, 1986) and previous studies. The upper and medial border, for the anterior portion of the BLA, was determined by the different cytoarchitecture of the BLA, which contrasted with the caudate-putamen and central nucleus of the amygdala; the posterior portion of BLA is adjacent to lateral ventricle. Laterally the BLA is bordered by the external capsule.

For vSub, only the pyramidal cell layers, where most parvalbumin interneurons were located, were counted. The division between vSub and CA1 subregions of the hippocampus was defined by an abrupt widening of the pyramidal layer. And the border between the vSub and presubiculum cortex was defined by the sharp transition to the smaller cells of the presubiculum. The borders of the hilus of the ventral DG were defined by the inner edge of the granule cell layer of DG, and pyramidal and molecular cell layers of CA3 (West et al., 1991).

### Data Analysis and Statistics

Estimates of neuron population were determined by the software-driven optical fractionator. For the vSub and ventral DG, a 2-way ANOVA was used to determine main effects and interaction of MAM and peripubertal diazepam administration; 1-way ANOVA followed by Bonferroni posthoc tests were used to determine difference among groups. For the BLA, difference between Sal and MAM rats was determined by Student’s *t* test. All statistical tests were performed using SPSS (IBM Software). Data were represented as mean ± SEM.

## Results

Loss of parvalbumin interneurons in vSub and DG of the hippocampus of MAM rats was attenuated by peripubertal administration of diazepam

**Figure 1.**
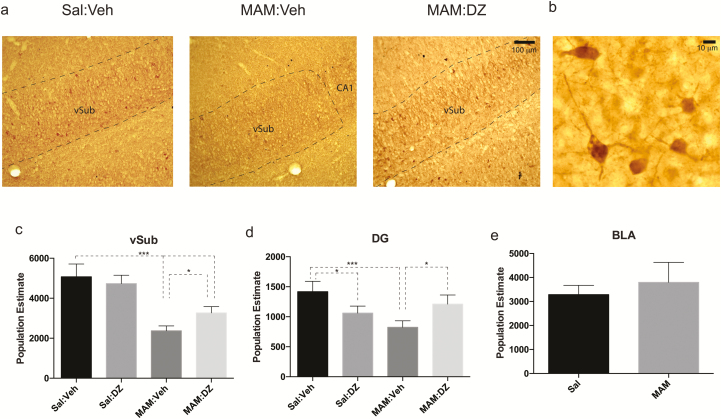
The loss of parvalbumin interneurons in methylazoxymethanol acetate (MAM) rats was attenuated by peripubertal diazepam administration. (a) Images of ventral subiculum (vSub) from saline (Sal), MAM, and MAM: diazepam (DZ) animals; scale bar = 100 μm. Boundaries of the counting area, pyramidal cell layer of vSub, are delineated by dotted lines. (b) Example image of parvalbumin interneurons. Arrows point to parvalbumin cells in focus; scale bar = 10 μm. (c) MAM rats exhibited a significantly lower number of parvalbumin interneurons in the vSub; however, MAM rats with peripubertal administration of diazepam (MAM:DZ) showed a significantly higher number of parvalbumin interneurons in the vSub compared with MAM:Veh rats, although the number is still significantly lower compared with Sal:Veh and Sal:DZ rats. *P<. 05; ***P<. 001. (d) In the hilus of the ventral DG, MAM:Veh rats showed a significantly lower number of parvalbmin interneurons than Sal:Veh, which was significantly attenuated in the MAM:DZ rats. Sal:DZ rats showed a significantly lower PV number than Sal:Veh rats. (e) The number of parvalbumin interneurons in the basolateral amygdala (BLA) did not differ significantly between Sal and MAM rats.

The average section thickness for all animals was 20.0±0.04 µm, accounting for a shrinkage of 67% ± 1%, with no significant difference among all groups. Parvalbumin interneurons of various sizes were observed, similar with previous findings (Kemppainen and Pitkänen, 2000). In the vSub, an average of 5072±319 parvalbumin interneurons was counted in control animals (Sal:Veh, n=4) Two-way ANOVA revealed a significant effect of MAM (F_1,13_=103.376, *P*<.001) and a significant interaction of MAM and diazepam treatment (F_1,13_=9.017, *P*=.01). Given this significant interaction, 1-way ANOVA was used to determine differences among groups. Peripubertal administration of diazepam did not significantly influence the number of parvalbumin interneurons in Sal animals (Sal:DZ, 4733±210, n=4). However, in MAM:Veh rats, the number of parvalbumin interneurons was significantly less (n=5, 2375±109, 1-way ANOVA, F_3,13_=40.092, *P<*. 001; Bonferroni posthoc test, MAM:Veh vs Sal:Veh, *P<*. 001). MAM rats with peripubertal administration of diazepam had significantly more parvalbumin interneurons compared with MAM:Veh (MAM:DZ, n=4, 3355±173, *P=*.047, Bonferroni posthoc test); however, this number was still significantly lower compared with Sal rats (MAM:DZ vs Sal:Veh, *P<*. 001) (Figure 1c).

For comparison, parvalbumin interneurons in the hilus of the ventral DG were also counted. Two-way ANOVA revealed a significant interaction (F_1,12_=27.97, *P=*.0002) and a significant effect of MAM (F_1,12_=9.934, *P=*.0083, n=4 for all groups). Consistent with previous studies (Gill and Grace, 2014), MAM:Veh rats exhibited significantly fewer parvalbumin interneurons (824±54) than Sal:Veh rats (1417±87). MAM rats with peripubertal diazepam showed significantly more parvalbumin interneurons than MAM:Veh (MAM:DZ, 1211±76, *P=*.01, Bonferroni posthoc following 1-way ANOVA), which was not significantly different from Sal:Veh and Sal:DZ rats (1060±59) (Figure 1d).

### MAM Rats Did Not Exhibit Loss of Parvalbumin Interneurons in the BLA

Shrinkage of sections throughout BLA of all animals was 64% ± 1%, rendering an average thickness of sections after shrinkage of 20.2±0.6 µm. The number of parvalbumin interneurons averaged 3283±175 in Sal rats (n=5), which was similar to that reported previously (Rostkowski et al., 2009). We did not observe significant differences between Sal and MAM rats in the number of parvalbumin neurons (MAM, 3797±372, n=5, t_8_=-1.247, *P*>.05, Student *t* test) (Figure 1e).

## Discussion

Using an unbiased stereological method, we observed a decrease in the number of parvalbumin positive interneurons in the vSub and ventral DG of the hippocampus of rats with the MAM developmental disruption model of schizophrenia, consistent with previous findings (Penschuck et al., 2006; Lodge et al., 2009; Chen et al., 2014; Gill and Grace, 2014). The decrease in parvalbumin immunostaining in the vSub and ventral DG is consistent with our previous studies (Lodge et al., 2009; Gill and Grace, 2014). Indeed, MAM-E17 treatment exerts a greater impact on parvalbumin interneurons in the ventral hippocampus. The hilus of the ventral DG was reported to show a reduced level of parvalbumin expression paralleled by a reduced number of parvalbumin cells; in contrast, a reduced parvalbumin expression was not accompanied by loss of parvalbumin cells in the dorsal hippocampus (Gill and Grace, 2014). Although we have not ruled out the possibility that our data reflect a loss of PV protein levels rather than a loss of PV neurons in the vSub, the large decrease in parvalbumin immunostaining highly suggests loss of parvalbumin interneurons in this region. Although peripubertal diazepam significantly increased parvalbumin interneurons in MAM rats in both vSub and ventral DG, the magnitude of the impact was different. MAM rats with peripubertal diazepam exhibited significantly lower number of parvalbumin interneurons in the vSub than controls; however, in the ventral DG, the number is not significantly different from controls. It is not clear why there was a small but significant (*P=*.02) decrease in the number of parvalbumin interneurons in the Sal:DZ rats compared with Sal:Veh, Nonetheless, peripubertal diazepam effectively attenuated loss of parvalbumin interneurons in both regions of the ventral hippocampus of MAM rats.

In contrast to the marked reduction of parvalbumin interneuron population in the ventral hippocampus of MAM rats, we did not observe any significant difference in the number of parvalbumin interneurons in the BLA. The specific impact of MAM-E17 on the hippocampus could be due to different prenatal development trajectories of the hippocampus and BLA. The peak of cell proliferation in the hippocampus is around E16 to E18 (Bayer, 1980), but BLA is actively developing between E14 and E16 (Berdel et al., 1997). As a mitotoxin, the effect of MAM in disrupting cell division lasts 12 to 24 hours after injection, when the hippocampus is actively developing but the BLA has already undergone development. Moreover, whether parvalbumin interneurons in the BLA of schizophrenia patients are altered is still unclear. Some early findings had suggested reduced GABAergic signaling in the BLA, but no recent work has focused on the GABAergic system in the BLA (Benes, 2010). Reduction in parvalbumin expression in the BLA has been observed in some animal models of schizophrenia (Romon et al., 2011; Nakamura et al., 2015) but not others (Pollard et al., 2012).

Parvalbumin interneurons, especially those in the hippocampus, are sensitive to stress. Hippocampal parvalbumin neurons were reduced in both social defeat and chronic mild stress models (Czeh et al, 2015; Hu et al, 2010). A hyperresponsivity to stress was observed in MAM rats during the peripubertal period; for example, enhanced baseline level of anxiety, increased vocalization to footshock, and lack of accommodation of the corticosterone response to chronic stress (Du and Grace, 2013; Zimmerman et al., 2013). This hyperresponsivity to stress might cause these rats to exhibit stress responses to normal housing conditions, which could damage parvalbumin interneurons in the hippocampus secondary to amygdala activation. The density of parvalbumin interneurons is already lower in MAM rats at PD25 (Gill and Grace, 2014), which could explain the incomplete reversal of the reduced parvalbumin interneuron numbers in MAM rats with peripubertal administration of diazepam on PD 31 to 40. However, by alleviating the hyperresponsivity to stress, this treatment prevents further loss of parvalbumin interneurons. Furthermore, as reported in previous studies, peripubertal administration of diazepam decreased the hyperdopaminergic state and the heightened anxiety levels in MAM rats (Du and Grace, 2013, 2016), which is probably contributed by the attenuated loss of parvalbumin interneurons. Nonetheless, this treatment has some efficacy to protect parvalbumin interneurons and by doing so, appears to keep the system beneath a threshold level of activation such that the cascade of events that is proposed to lead to the emergence of hyperdopaminergic state in MAM rats will not be triggered. Other treatments early in life, such as antipsychotics (Piontkewitz et al., 2012) or antioxidants (Cabungcal et al., 2014), have also been shown to effectively protect parvalbumin interneurons and prevent other schizophrenia-like symptoms from emerging in developmental disruption models of schizophrenia. Therefore, we propose that controlling the hyperresponsivity to stress during the premorbid period in subjects at risk of schizophrenia, either pharmacologically or nonpharmacologically, may be an effective mean to prevent development of schizophrenia later in life.

## Statement of Interest

A.A.G. has received funds from Johnson & Johnson, Lundbeck, Pfizer, GSK, Merck, Takeda, Dainippon Sumitomo, Otsuka, Lilly, Roche, Asubio, Abbott, Autofony, Janssen, and Alkermes. Y.D. has no conflicts of interests.
